# 

**Published:** 1974-01

**Authors:** 


OCT 24 1975

OCT 21 1975

The
Bristol Medico-Chirurgical Journal
(Incorporating the Medical Journal of the South-West)
Established 1883

Vol. 89 (i) JANUARY 1974 No. 329

### BRISTOL MEDICO-CHIRURGICAL JOURNAL

Editor: N. J. Brown, M.B., F.R.C.P., F.R.C.Path.

Published Quarterly

Annual Subscription £2 Post Free

### BRISTOL MEDICO-CHIRURGICAL SOCIETY

**President 1973-74: Dr. R. F. Barbour**

**President-elect: Dr. J. Apley**

**Honorary Secretary : Dr. I. S. Bailey, 7 Percival Road, Bristol 8**

**Honorary Treasurer : Dr. Frank Ross, Bristol Royal Infirmary, Bristol 2**

The Society was founded in 1874. In 1883 publication of the Journal began and has continued quarterly ever since then. During the years 1949 to 1962 it appeared under the title of "The Medical Journal of the South West".

### THE BRISTOL MEDICO-CHIRURGICAL JOURNAL

### Editorial Committee

| | |
|---|---|
| Editor | Dr. N. J. Brown, Southmead Hospital, Bristol. |
| Assistant Editor | Dr. D. S. Reeves. |
| Members | Dr. Rhys Davies.<br>Dr. M. B. Lennard.<br>Professor R. C. Wofinden.<br>Dr. D. W. Wright.<br>The Hon. Treasurer and Hon. Secretary of the Society. |
| Business Manager | Dr. P. J. F. Baskett, Frenchay Hospital, Bristol. |
| Secretary | Mr. B. P. Jones, The Library, Medical School, University Walk, Bristol BS8 1TD. |

### NOTICE TO CONTRIBUTORS

The Journal is especially concerned to provide a place for the recording of medical thought, interests, and practice in and around Bristol. It therefore welcomes articles that, as well as being of immediate interest to members, will document the local and contemporary medical scene for our successors.

Original articles are invited provided that they have not been published elsewhere. References in the text should be by author's name, with year of publication in parenthesis; they should be listed at the end in alphabetical order, the name of each journal or book being given in full—not abbreviated—and the title of the article added. Illustrations should be supplied ready for reproduction. Line drawing should be in Indian ink on firm white paper or board. The author will be informed if it is found necessary to ask him to pay for the making of blocks.

The following contributions will be especially welcome: notes of forthcoming events, meetings held, degrees conferred, staff appointments, honours and public appointments, and other local news.

### The Medical Library

In 1890 the Bristol Medico-Chirurgical Society founded a medical library which in 1892 was combined with that of the Bristol Medical School. This became the University of Bristol Medical Library in 1925 and an agreement in that year confirmed the privilege of Members of the Society to use the University Medical Library.

### A Selection of Recent Additions to the Medical Library

GOLD, E. R. & N. R. BUTLER.: ABO haemolytic disease of the newborn. (Wright) Presented by John Wright & Sons Ltd.

GOODLIN, R. C.: Handbook of obstetrics & gynaecology. (Geron-X).

GRAHAME-SMITH, D. G.: The carcinoid syndrome. (Heinemann)

GRAY, S. W. & J. E. SKANDALAKIS.: Embryology for surgeons. (Saunders)

GRUNDMANN, E. & H. TULINIUS eds.: Current problems in the epidemiology of cancer, lymphomas & leukemias. (Springer)

HALLER, J. A. & J. L. TALBERT.: Surgical emergencies in the newborn. (Lea & Febiger)

HARVEY, S & A. HALES-TOOKE.: Play in hospital. (Faber)

HAUSER, M. M.: The economics of medical care. (Allen & Unwin)

HEATON, K. W.: Bile salts in health & disease. (Churchill Livingstone) Presented by the Author.

HIROHATA, K. & K. MORIMOTO.: Ultrastructure of bone & joint disease. (Excerpta Medica)

HOFFBRAND, A. V. & S. M. LEWIS.: Tutorials in postgraduate medicine—Haematology. (Heinemann)

HOLLING, H. E.: Peripheral vascular diseases: diagnosis & managment. (Lippincott).

HOLMES, L. B. et al.: Mental retardation: an atlas of diseases with associated physical abnormalities. (Macmillan)

HOPKINS, P. ed.: Patient-centred medicine. (Regional Doctor Publns. Ltd.)

HOWE, G. M.: Man, environment & disease in Britain: a medical geography of Britain through the ages. (Barnes & Noble/David & Charles)

HURLEY, J. V.: Acute inflammation. (Churchill Livingstone)

ILLIS, L. S. & J. V. T. GOSLING.: Herpes simplex encephalitis. (Scientechnica). Presented by Scientechnica.

IZAK, G. & S. M. LEWIS eds.: Modern concepts in hematology. (Acad. Press)

JAFFE, H. L.: Metabolic, degenerative & inflammatory diseases of bones & joints. (Lea & Febiger)

JANZEN, R. et al.: Pain: principles, pharmacology, therapy. (Thieme)

JENSEN, K. G.: Peptic ulcer: genetic & epidemiological aspects based on twin studies. (Munksgaard)

KAKKAR, V. V. & A. J. JOUHAR eds.: Thromboembolism: diagnosis & treatment. (Churchill Livingstone)

KUCERS, A.: The use of antibiotics: a comprehensive review with clinical emphasis. (Heinemann)

LADER, M. & I. MARKS.: Clinical anxiety. (Heinemann)

LOCATCHER-KHORAZO, D. & B. C. SEEGAL.: Microbiology of the eye. (Mosby)

LUDWIG, J.: Current methods of autopsy practice. (Saunders)

M. D. ANDERSON HOSPITAL & TUMOR INSTITUTE.: Breast cancer: early and late. (Year Bk.)

McLAREN, D. S.: Nutrition & its disorders. (Churchill Livingstone)

MILNE, J. A.: An introduction to the diagnostic histopathology of the skin. (Arnold)

OLIVER, M. F. et al. eds.: Effect of acute ischaemia on myocardial function. (Churchill Livingstone)

PERKINS, E. S. & J. M. DOBREE.: The differential diagnosis of fundus conditions. (Kimpton)

PIA, H. W. et al.: Modern aspects of neurosurgery. (Excerpta Medica)

POWLEY, P.: Trauma surgery, excepting bones & joints. (Wright) Presented by John Wright & Sons Ltd.

PRESTON, S. M. et al.: Causes of death: life tables for national populations. (Seminar Press)

REIMANN, H. A.: The pneumonias. (Hilger)

ROSE, J. ed.: Computers in medicine. (Wright) Presented by John Wright & Sons Ltd.

RUSSELL, J. G. B.: Radiology in obstetrics & antenatal paediatrics. (Butterworths)

SAEGESSER, F. & J. PETTAVEI eds.: Surgical oncology. (Huber)

SCHREIBER, P.: Anesthesia equipment: performance, classification & safety.

SHAFRIR, E. ed.: Impact of insulin on metabolic pathways. (Acad. Press)

SHEENAN, H. L. & J. B. LYNCH.: Pathology of toxaemia of pregnancy. (Churchill Livingstone).

SMALL, W. P. & U. KRAUSE.: An introduction to clinical research. (Churchill Livingstone)

SPEERT, H.: Iconographia gyniatrica: a pictorial history of gynecology and obstetrics. (Davis)

STAMEY, T. A.: Urinary infections. (Williams & Wilkins)

SUTTON, N. G.: Injuries of the spinal cord: the management of paraplegia & tetraplegia. (Butterworths)

VOLK, B. W. & S. M. ARONSON eds.: Sphingolipids, sphingolipidoses & allied disorders. (Plenum Press)

WALDENSTROM, J.: Diagnosis & treatment of multiple myeloma. (Grune & Stratton)

WANNAMAKER, L. W. & J. M. MATSEN, eds.: Streptococci and streptococcal diseases. (Acad. Press)

WEED, L.: Medical records, medical education & patient care. (Year Bk.)

WESTS, G.P.: Rabies in animals & man. (David & Charles)

WILLIAMS, G.: A colour atlas of renal diseases. (Wolfe)

WILLS, M. R.: The biochemical consequences of chronic renal failure. (Harvey Miller & Medcalf)

WOODS, C. G.: Diagnostic orthopaedic pathology. (Blackwell)

ZUBIN, J. & A. M. FREEDMAN eds.: Psychopathology of adolescence. (Grune & Stratton)

### Bristol Medico-Chirurgical Society

The Annual General Meeting of the Society was held in the Medical School on 10th October 1973. Dr. N. J. Brown showed pathological specimens and Dr. G. R. Airth demonstrated skiagrams. Mr. B. P. Jones arranged a display of medical books. The President, Dr. James Macrae, resigned the Chair to his successor, Dr. R. F. Barbour, who delivered his Inaugural Address on the subject of the history of psychiatry in Bristol. This address, together with other appropriate historical papers will appear in the July 1975 number of the Journal which will be a special issue celebrating the Centenary Year of the Society. A vote of thanks to the President for his address was proposed by Mr. G. M. Fitzgibbon and a vote of thanks to the retiring president by Dr. Alan Roberts. Dr. Lumsden Walker proposed the nomination of Dr. John Apley as President-elect and this was warmly approved. After election of other officers, Annual Reports were received from the Hon. Treasurer, Hon. Secretary and Hon. Editor of the Journal. The Editor reported that the financial difficulties that had led to publication of the Journal falling into arrears had been overcome and hoped that it would be possible to achieve punctual publication again next year. He was pleased to report that there was no lack of material and hoped that the steady flow of papers from local authors would continue.

The Clinical Meeting was held at Glenside Hospital on 14th November. Video-tape Clinical Cases for discussion were introduced by Dr. H. G. Morgan and after supper a discussion on The Past, Present and Future was introduced by Dr. H. Temple Phillips, Dr. M. Nicholas and Dr. D. F. Early.

On 12th December the third meeting of the session was held at the Royal Infirmary and took the form of a Symposium on 'Light in Dermatology' presented by Doctors R. P. Warin, R. R. M. Harman, R. D. G. Peachey and J. L. Burton.

### Notes and News

**University of Bristol**
M.D.—J. D. Anderson
Ph.D (Faculty of Medicine)—E. P. Beck, S. A. R. Choudhury, H. A. Hossain.

**M.R.C.Path**
D. J. Goldie, T. J. Hamblin.

